# Impact of Gluten-Friendly Bread on the Metabolism and Function of *In Vitro* Gut Microbiota in Healthy Human and Coeliac Subjects

**DOI:** 10.1371/journal.pone.0162770

**Published:** 2016-09-15

**Authors:** Antonio Bevilacqua, Adele Costabile, Triana Bergillos-Meca, Isidro Gonzalez, Loretta Landriscina, Emanuela Ciuffreda, Paola D’Agnello, Maria Rosaria Corbo, Milena Sinigaglia, Carmela Lamacchia

**Affiliations:** 1 Department of the Science of Agriculture, Food and Environment, University of Foggia, Via Foggia, Italy; 2 Department of Food and Nutritional Sciences, The University of Reading, Reading, United Kingdom; University of Palermo, ITALY

## Abstract

The main aim of this paper was to assess the *in vitro* response of healthy and coeliac human faecal microbiota to gluten-friendly bread (GFB). Thus, GFB and control bread (CB) were fermented with faecal microbiota in pH-controlled batch cultures. The effects on the major groups of microbiota were monitored over 48 h incubations by fluorescence *in situ* hybridisation. Short-chain fatty acids (SCFAs) were measured by high-performance liquid chromatography (HPLC). Furthermore, the death kinetics of *Lactobacillus acidophilus*, *Bifidobacterium animalis* subsp. *lactis*, *Staphylococcus aureus*, and *Salmonella* Typhimurium in a saline solution supplemented with GFB or CB were also assessed. The experiments in saline solution pinpointed that GFB prolonged the survival of *L*. *acidophilus* and exerted an antibacterial effect towards *S*. *aureus* and *S*. Typhimurium. Moreover, GFB modulated the intestinal microbiota *in vitro*, promoting changes in lactobacilli and bifidobacteria members in coeliac subjects. A final multivariate approach combining both viable counts and metabolites suggested that GFB could beneficially modulate the coeliac gut microbiome; however, human studies are needed to prove its efficacy.

## Introduction

Coeliac disease is a chronic immune-mediated enteropathy triggered by the ingestion of gluten in HLA-DQ2- or HLA-DQ8-positive people. Approximately 30% of the general population carries the HLA-DQ2/8 coeliac disease susceptibility genes; however, only 2–5% of these individuals will go on to develop coeliac disease, suggesting that additional environmental factors contribute to the disease development [1]. Many authors have reported that coeliac people suffer from an altered composition of gut microbiota [2,3], such as lower levels of *Bifidobacterium* spp. and *Lactobacillus* spp. [4,5]; higher levels of *Bacteroides*, *Escherichia coli*, *Staphylococcus* and *Clostridium* [4,6–8]; and an altered profile of short-chain fatty acids (SCFAs) [4,9]. The link between an altered gut microbiota composition and disease is a matter of debate, as some authors have suggested that this change could be both an effect of disease and the cause of some symptoms [10].

To date, the only treatment for coeliac patients is the complete lifelong exclusion of gluten from the diet, but a gluten-free diet does not completely restore healthy microbiota profiles [11]. An alternative to the exclusion of gluten is its detoxification without affecting the technological performance of the flour and dough. Some approaches for gluten detoxification have been proposed and studied in the past, e.g. the use of protease produced by lactic acid bacteria [12] or the transdamidation [13]. Recently, we developed a new and innovative method to detoxify gluten proteins from cereal grains with the purpose of combining the nutritional and technological properties of wheat proteins with safety for coeliac and gluten-sensitive patients [14,15]. This innovation is usually referred to as “gluten friendly” and relies on the application of microwave energy for a few seconds to hydrated wheat kernels before milling to reach a high temperature for a short amount of time and induce a structural change in gluten proteins [16]. This modification abolishes the antigenic capacity of gluten [16] and reduces *in vitro* the immunogenicity of the most common epitopes involved in coeliac disease [17], without compromising the nutritional and technological properties necessary to process semolina in pasta and flours in bread and other baked goods [16]. Additionally, the technology has been further improved [18].

Scaling up a new technology is a complex process because many issues must be addressed. For a gluten-friendly approach, we have only assessed the effect of microwave on gluten proteins [16], and the industrial scale-up is in progress. This paper addresses the effect of gluten-friendly bread (GFB) on the gut microbiota composition through two intermediate steps:

1. Assessing the effect on certain foodborne strains (both probiotic and pathogenic) to pinpoint whether the addition of GFB modifies the survival of these selected targets under strict controlled conditions. This step was necessary to select and/or design an experiment evaluating the effects of GFB in a complex system;
2. Investigating the effect of GFB on faecal human microbiota in healthy and coeliac subjects in a pH-controlled, stirred, batch-culture fermentation system that is reflective of the environmental conditions of the distal region of the human large intestine, with a focus on the viable count of certain microbial groups and the production of SCFAs.

## Materials and Methods

### Raw materials and microwave treatment

The Casillo group S.p.a (Corato, Italy) supplied the wheat kernels (mixtures of soft wheat Canadian grains; the exact composition of the mixtures was not specified). Flour treated with microwaves was called gluten-friendly flour (GFF) and was obtained by milling the microwave-treated caryopses [18]. One hundred grams of cleaned wheat grains were dampened until reaching 15–18% humidity, which was measured by a halogen thermal balance (Mettler Toledo, HB43-S, Switzerland), and subjected to rapid heating via microwaves (Delonghi, Italy, approximately 1 min. between 1000 and 750 watts), followed by slow evaporation of the water. The rapid heating and subsequent slow evaporation of the water was repeated until reaching a final temperature of 80–90°C, which was measured by a thermal camera (Fluke, i 20 model, Italy), and a moisture degree of 13–13.5% in the wheat grains.

After microwave treatment, the wheat kernels were cooled and dried at room temperature (24°C) for 12–24 h and then ground using an automatic laboratory mill MCKA (Bühler AG, Azwil, Switzerland, diameter of grid 118–180 μm). The flour produced by milling caryopses that had not been treated with microwaves was called control flour (CF). The particle size of the GFF and the CF used was in the range of 100 to 200 μm.

### Bread production and digestion

Bread was produced by using either CF (control bread, CB) or GFF (GFB) according to the Chorleywood Bread Process in the Food Processing Centre of the Department of Food and Nutritional Sciences at the University of Reading (UK). Bread was prepared as follows: flour, 100 g; water, 66 mL; baker’s yeast, 1.33 g; and salt, 1 g. Allinson Easy Bake Yeast (UK) has been used as a dried yeast with the bread improver Ascorbic Acid (Vitamin C).

### Simulated *in vitro* human digestion

Bread was digested in *vitro* under appropriate conditions according to the procedures described by Maccaferri et al. [19] in order to mimic mouth, stomach and intestine’s condition. The only modification to the method described by Macaferri et al. [19] was to not apply any form of dialysis to the samples.

### *In vitro* experiments

#### Microorganisms

*Lactobacillus acidophilus* La-5, *Bifidobacterium animalis* subsp. *lactis* Bb-02, *Salmonella* Typhimurium and *Staphylococcus*. *aureus* were used throughout this research. *L*. *acidophilus* and *B*. *animalis* were purchased from Chr. Hansen and stored at -20°C in MRS broth (Oxoid, Milan, Italy) supplemented with 33% sterile glycerol (J.T. Baker, Milan). The pathogens were food-borne isolates belonging to the culture collection of the Laboratory of Predictive Microbiology, University of Foggia; the strains were stored at 4°C on tryptone soya agar slants (Oxoid).

Before each assay, the microorganisms were grown at 37°C for 24 h in the optimal media (MRS broth or TSB broth); the cultures were centrifuged two times at 1000 g for 10 min, and the cells were suspended in sterile distilled water.

#### Samples and microbiological analyses

Two different sets of experiments were performed, as reported in Table 1. Aliquots of saline solution (0.9% NaCl) (50 mL) were supplemented with different amounts of dried CB or dried GFB and inoculated to approximately 8 log CFU mL^-1^; the samples were periodically analysed to assess the viable count by plating on MRS agar (*L*. *acidophilus* and *B*. *animalis*) or TSA (*Salmonella* sp., *S*. *aureus*) and incubating at 37°C for 2–4 days. *L*. *acidophilus* and *B*. *animalis* were assessed under anaerobic conditions.

**Table 1:** Conditions used to assess the effect of control bread and gluten friendly bread towards *L*. *acidophilus*, *B*. *animalis*, *Salmonella* sp. and *S*. *aureus* in saline solution.

Experiments	Targets	Samples	Duration
**Death kinetics**	*L*. *acidophilus B*. *animalis*	Saline solution and samples supplemented with 0.4 or 0.8 g L^-1^ of either CB or GFB*	7 days (viable count every 6–10 h)
**Effect of concentration**	*L*. *acidophilus B*. *animalis*	Saline solution and samples supplemented with 0.8 or 5.0 g L^-1^ of either CB or GFB	24 h
**Pathogens**	*Salmonella* Typhimurium *S*. *aureus*	Saline solution and samples supplemented with 0.2, 0.4, or 0.8 g L^-1^ of either CB or GFB	7 days (viable count after 1 and 7 days)

*CB, control bread; GFB, gluten friendly bread.

#### Modelling and statistics

The experiments were performed on two different batches in duplicate (n = 2).

The results of the viable count were fitted using the Weibull equation in the log-linear form [20]:

logN=logN0−(tδ)p

where log N is the cell count over time (log CFU mL^-1^); log N_0_ is the initial cell count; δ is the first reduction time (day), i.e., the time to attain a decrease in cell count of 1 log CFU mL^-1^; and p is the shape parameter (p>1, downward curve; p<1, upward curve).

The data were also modelled through Weibull equation, as modified by Bevilacqua et al. [21], to evaluate the death time of the population.

The fitting parameters of the Weibull equation, as well as the viable counts, were analysed by one-way analysis of variance (ANOVA) and Tukey’s test as the *post-hoc* comparison test (P<0.05). Statistics were performed in Statistica software for Windows (StatSoft, Tulsa, OK).

### Batch culture fermentation

#### Collection and faecal sample preparation

Faecal samples were obtained from three healthy human volunteers (two males, one female; age 30 to 38 years; BMI, body mass index of 18.5–25) who were free of known metabolic and gastrointestinal diseases (e.g., diabetes, ulcerative colitis, Crohn’s disease, irritable bowel syndrome, peptic ulcers and cancer). All healthy faecal donors had the experimental procedure explained to them and were given the opportunity to ask questions. All donors then provided written informed consent for the use of their faeces in the study, and a standard questionnaire to collect information regarding the health status, drug use, clinical anamnesis, and lifestyle was administered before the donor was asked to provide a faecal sample. For the coeliac donors (two females, one male; age 30 to 38 years; BMI of 18.5–25), written informed consent was also obtained, and the *in vitro* study was approved by the University of Reading Research Ethics Committee (UREC 15/20). All faecal samples from healthy and coeliac donors were collected on site, stored in an anaerobic cabinet (10% H_2_, 10% CO_2_ and 80% N_2_) and used within a maximum of 15 min after collection. The samples were diluted 1/10 wt/vol in anaerobic phosphate-buffered saline (PBS, 0.1 M phosphate buffer solution, pH 7.4) and homogenised (Stomacher 400, Seward, West Sussex, UK) for 2 min (460 paddle-beats/min). To maintain the anaerobic conditions, the PBS was maintained in anaerobic cabins until the time of use. The resulting faecal slurries from each individual were used to inoculate the batch-culture systems.

#### Batch culture fermentations

Previously sterilized batch culture fermentation vessels (280 mL working volume) were filled with 45 mL of sterile complex colonic model growth medium. The composition of this medium included peptone water (5 g L^-1^), yeast extract (4.5 g L^-1^), starch (5 g L^-1^), tryptone (5 g L^-1^), NaCl (4.5 g L^-1^), KCl (4.5 g L^-1^), mucin (4 g L^-1^), casein (3 g L^-1^), pectin (2 g L^-1^), xylan (2 g L^-1^), arabinogalactan (2 g L^-1^) and inulin (1 g L^-1^) [22]. All media and chemicals were purchased from Oxoid and Sigma. Then, the vessels were connected to a circulating water bath at 37°C and sparged with O_2_-free N_2_ gas overnight to attain anaerobic conditions. The pH was adjusted to between 6.7 and 6.9 using pH meter controllers with NaOH or HCl (Electrolab260; Electrolab Ltd., Tewkesbury, UK), and 5 mL of faecal slurry was then inoculated in each vessel. In total, eighteen vessels were prepared and supplemented with 1 mL of CB or GFB digesta (3 vessels per type of donor, including a negative control, i.e., a sample containing faecal slurry but without bread, and samples with CB and GFB). The batch cultures were run for 48 h, and 5 mL of the samples were removed at 0, 6, 24 and 48 h for analysis of bacterial populations by fluorescence *in situ* hybridisation (FISH) and SCFA analysis by high-performance liquid chromatography (HPLC).

#### Enumeration of bacterial populations by fluorescence in situ hybridisation (FISH)

FISH was performed as described by Costabile et al. [23]. A 375 μL aliquot of the batch culture samples was fixed in three volumes of ice-cold 4% (w/v) paraformaldehyde for 4 h at 4°C, centrifuged at 13,000 g for 5 min and washed twice in 1 mL of sterile PBS. The cells were again pelleted by centrifugation and re-suspended in 150 μL of sterile PBS, to which 150 μL of ethanol was added. The samples were then mixed and stored at -20°C until used.

All probes were synthesised by Sigma-Aldrich. The following bacterial groups were identified using synthetic oligonucleotide probes that target specific regions of the 16S ribosomal RNA molecule, labelled with the fluorescent dye Cy3: *Clostridium hystolyticum* clusters I/II (Chis150, TTATGCGGTATTAATCTYCCTTT) [24], *Lactobacillus*/*Enterococcus* spp. (Lab158, GGTATTAGCAYCTGTTTCCA) [25], *Clostridium* clusters XIVa+b (Erec482, GCTTCTTAGTCARGTACCG) [24], *Bacteroides*/*Prevotella* group (Bac303, CCAATGTGGGGGACCTT) [26], *Bifidobacterium* spp. (Bif164, CATCCGGCATTACCACCC) [27] and Eub338 I-II-III (GCTGCCTCCCGTAGGAGT, GCAGCCACCCGTAGGTGT, GCTGCCACCCGTAGGTGT) [28].

#### Short-chain fatty acid (SCFA) analysis

Samples were taken from the batch culture vessels at each time point, and cell-free culture supernatants were obtained by centrifuging 1 mL at 13000 x g for 10 min, followed by filter sterilisation (0.2 μm Acrodisc^®^ syringe filters with a hydrophilic polyvinylidene fluoride (PVDF) membrane, 13 mm; Pall Corporation) to remove particulate matter. The SCFA content was quantified on an ion exclusion HPLC (LaChrom Merck Hitachi, Poole, Dorset, UK) instrument equipped with a pump (L-7100), RI detector (L-7490) and autosampler (L-7200). Samples (20 μL) were injected into the HPLC at a flow rate of 0.5 mL min^-1^ with a prepacked Rezex ROA–Organic Acid H+ 80% (300 x 7.8 mm) column at 84°C and a detector wavelength of 210 nm. H_2_SO_4_ (2.5 mM) was used as the eluent, and the organic acids (lactic, acetic, propionic and butyric) were calibrated against standards at concentrations of 12.5, 25, 50, 75 and 100 mM. An internal standard of 2-ethylbutyric acid (20 mM) was included in the samples and external standards. All chemicals were provided from Sigma-Aldrich.

#### Statistical analysis

The results from the FISH and SCFA analyses were standardized and reported as increases/decreases relative to t_0_ of the negative control (beginning of the experiment). The results were first analysed through one-way ANOVA and Tukey’s test using the homogeneous group approach [29]. The classical approach of one-way ANOVA and post-hoc offers an overview of the significant differences amongst different samples. However, it does not work well if the studied parameter does not show or possess well-defined statistical groups and is distributed in a continuous way and if each sample can be attributed to different statistical groups. When this type of distribution is observed in the results, the use of ANOVA by homogeneous groups is advisable: the parameters are organized in a table with different columns (homogeneous groups), and each column contains the samples belonging to the same homogeneous group. The novelty relies upon the fact that the same sample can be attributed to many groups.

Thereafter, the FISH and SCFA values were used as input data to run 3 different principal component analysis (PCA; for the results after 6, 24, and 48 h) experiments.

As a confirmatory experiment, a second standardization of the data was performed, i.e., for each sampling time, the results from healthy and coeliac donors supplemented with CB or GFB were reported as increases/decreases relative to the negative control at the same time, and the results were then analysed through one-way ANOVA. This type of modelling was used to exclude possible prebiotic activities of CB or GFB on both healthy and coeliac people.

## Results

### *In vitro* experiments

Table 2 reports the fitting parameters for *L*. *acidophilus*; the death kinetics generally showed a downward curve with a shape parameter of the Weibull equation >1. The supplementation of saline solution with either CB or GFB did not affect the shape of the curve. On the other hand, the type of bread exerted a significant effect on the death time of the bacterial population; in fact, the death time was prolonged from 67.46 (CB) to 80.53 (GFB) at 0.4 g L^-1^ and from 70.28 (CB) to 93.96 (GFB) at 0.8 g L^-1^.

**Table 2:** Fitting parameters of the Weibull equation for the death kinetics of *L*. *acidophilus* (mean values ± SE). For each parameter, the letters indicate significant differences (one-way ANOVA and Tukey’s test, P<0.05). CB, control bread; GFB, gluten-friendly bread.

Sample	log N_0_*	δ	p	d.t.	R
CB 0.4 g L^-1^	8.43±0.14A	17.99±0.90A	1.62±0.15A	67.46±2.06A	0.995
GFB 0.4 g L^-1^	8.38±0.13A	17.43±2.06A	1.40±0.14A	80.53±2.03B	0.994
CB 0.8 g L^-1^	8.19±0.12A	23.40±2.00B	1.94±0.20A	70.28±2.63A	0.993
GFB 0.8 g L^-1^	8.56±0.14A	17.57±2.70A	1.27±0.17A	93.96±4.00C	0.990

*log N_0_, initial cell count (log CFU mL^-1^); δ, first reduction time (h); p, shape parameter; d.t., death time (h).

**Fig 1:**
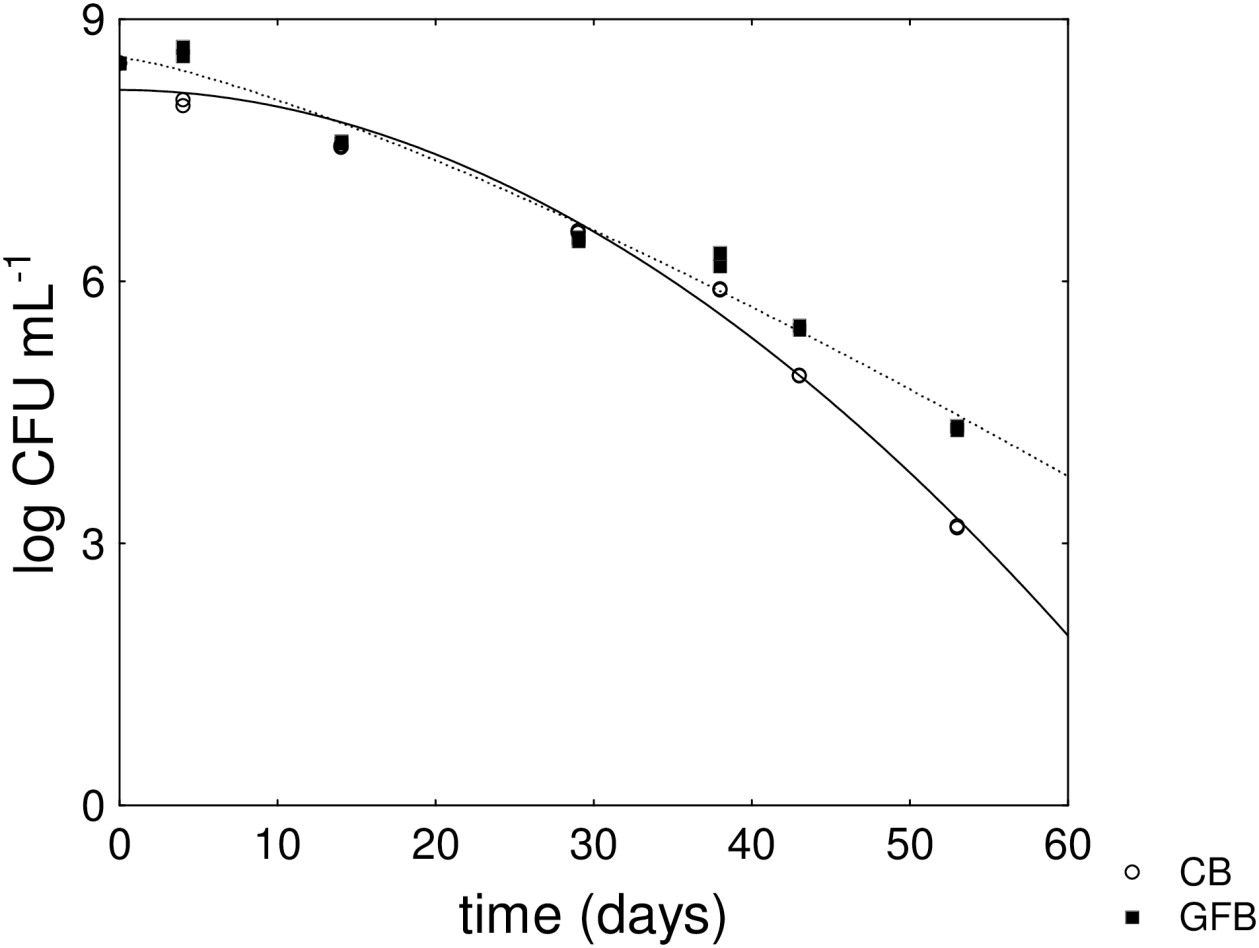
Death kinetics of *L*. *acidophilus* in a saline solution supplemented with either control (CB) or gluten-friendly bread (GFB) (0.8 g L^-1^). The lines represent the best fit through the Weibull equation.

The effect of GFB on the death time, but not on the shape parameter, was a consequence of a probable reduction of the death rate in the last part of the death curve, as suggested by the kinetics reported in Fig 1. The death kinetic of *B*. *animalis* subsp. *lactis* was not affected by the type of supplementation (S1 Table).

A second assay was run to determine whether the concentration of GFB could cause or exert a detrimental effect on both *L*. *acidophilus* and *B*. *animalis*; saline solution was supplemented with same the amount used in the first experiment (0.8 g L^-1^) and with a higher concentration (5.0 g L^-1^). The viable count was not affected by the concentration of bread digesta (S2 Table).

**Fig 2:**
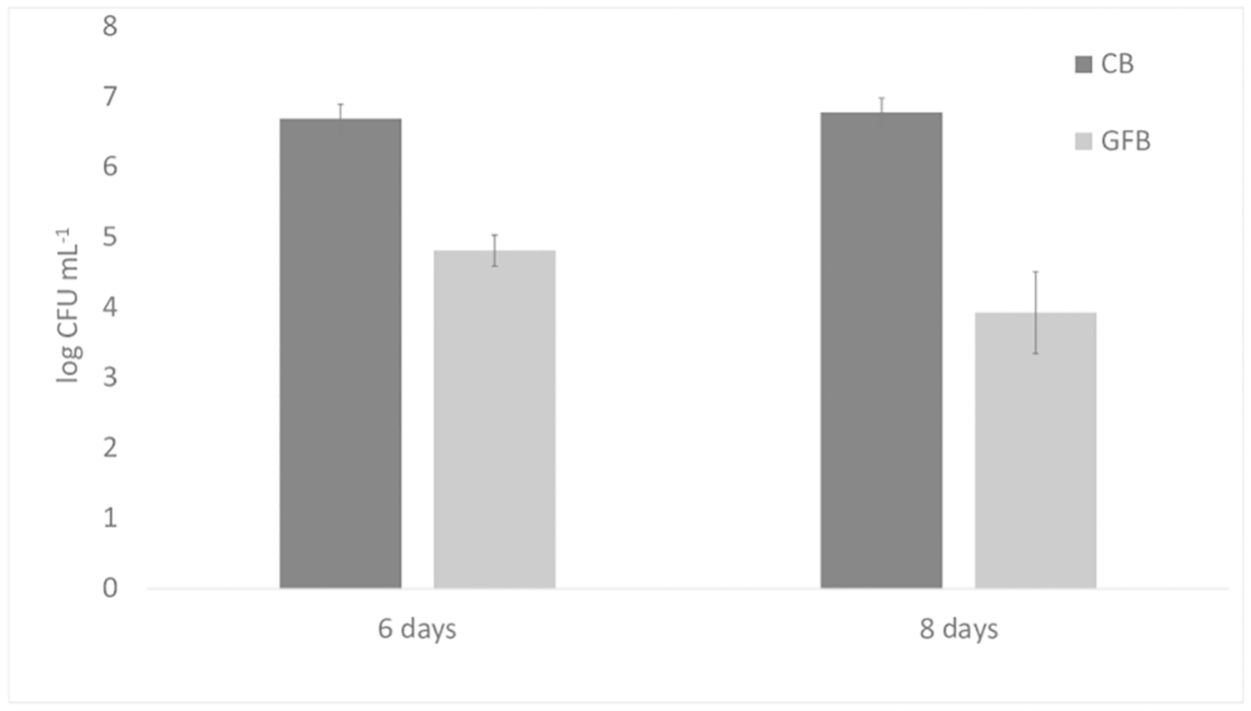
Viable count of *S*. *aureus* in a saline solution supplemented with either control (CB) or gluten-friendly bread (GFB) (0.2, 0.4 or 0.8 g L^-1^). Mean values ± standard deviation. The symbols “*” and “**” denote significant differences (one-way ANOVA and Tukey’s test).

**Fig 3:**
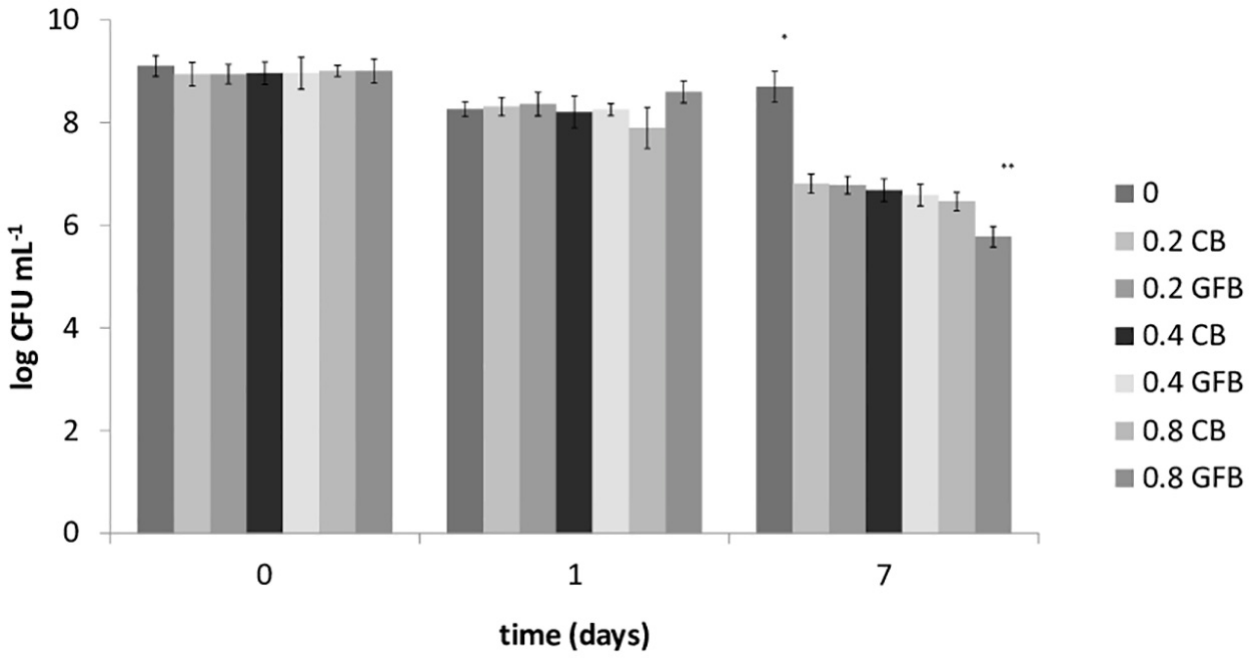
Viable count of *Salmonella typhimurium* in a saline solution supplemented with either control (CB) or gluten-friendly bread (GFB) (0.8 g L^-1^). Mean values ± standard deviation.

Finally, saline solution was inoculated with a Gram-positive or a Gram-negative pathogen (*S*. *aureus* and *Salmonella* Typhimurium); the results for *S*. *aureus* are shown in Fig 2. A significant difference was found for the sample supplemented with 0.8 g L^-1^ GFB, which showed a 1-log lower viable count than the sample supplemented with CB. *Salmonella* sp. experienced a 1.8- and 2.84-log reduction after 6 and 8 days, respectively (Fig 3).

### Changes in bacterial populations and the metabolic profile with *in vitro* batch culture fermentation: a one-way ANOVA approach

A second step was aimed at assessing the effect of both CB and GFB in batch culture fermentations. See S1 and S2 Figs for the raw data.

The results of the FISH and SCFA analyses were standardized to the negative control at time 0 (inoculation time) to exclude the variability due to the type of donor; thus, the results show the modification of the system compared to the beginning of the experiment and should be read as increases (positive values) or decreases (negative values). Moreover, each parameter was analysed through one-way ANOVA to pinpoint significant differences; the homogeneous group approach was used as an additional tool to uncover a possible trend through time.

Table 3 shows the results for bifidobacteria. The differences amongst the samples were not significant after 6 h or 24 h, but two statistical groups were recovered after 48 h, i.e., sample E (coeliac donor with CB) and the other samples. Sample E did not show a significant increase in the viable count of bifidobacteria (0.27-log increase) probably due to a detrimental effect exerted by bread on the microbiota. In the other samples, there was a 0.7–09 log increase, and sample F (coeliac donor with GFB) experienced a similar trend to that of the samples from healthy donors.

**Table 3:** One-way ANOVA (P<0.05) and homogeneous groups based on FISH data for bifidobacteria (enumerated as Bif164) after 6, 24 and 48 h of fermentation in pH-controlled batch culture systems. Increases/decreases refer to the inoculum of the negative control. Samples: A, negative control healthy donors; B, healthy donors + CB; C, healthy donors + GFB; D, negative control coeliac donors; E, coeliac donors + CB; F, coeliac donors + GFB. CB, control bread; GFB, gluten-friendly bread.

	FISH (log cells mL^-1^)	Homogeneous groups		
6 h		I	II	III
Sample				
E	0.15			
F	0.26			
D	0.49			
B	0.60			
A	0.63			
C	0.70			
**24 h**				
E	0.37			
F	0.49			
D	0.51			
A	0.72			
B	0.73			
C	0.91			
**48 h**				
E	0.27			
A	0.65			
F	0.68			
D	0.70			
B	0.72			
C	0.93			

Concerning the outputs for the *E*. *rectale–C*. *coccoides* group (enumerated as Erec482) and the *Bacteroides* spp.–*Prevotella* group (enumerated as Bac303), the statistics highlighted a continuous rather than discrete distribution of the samples, with 2–4 overlapping homogeneous groups depending on the time and the type of microorganisms present. The sample distribution of *Bacteroides/Prevotella* changed over time; however, the increase/decrease in the absolute values of the viable count (-0-33-0.26 log CFU mL^-1^) was slight (S3 Table).

Table 4 shows the effect of bread supplementation on the numbers of the *C*. *hystolyticum* group (Chis150). After 6 h, there was a continuous distribution of samples with 2 well-defined groups (first group containing samples A and B; second group containing sample E) and an intermediate class (samples C, D, and F). Thus, sample E (coeliac donor with CB) was not different from samples D and F (negative control and coeliac donor with GFB), although it was significantly different from the healthy donor samples. Samples F and D experienced a slight statistical shift towards sample C. This shift was not observed after 24 and 48 h, suggesting that this result should be confirmed with prolonged experiments and repeated supplementation.

**Table 4:** One-way ANOVA (P<0.05) and homogeneous groups based on fluorescence *in situ* hybridization (FISH) data for the *C*. *hystolyticum* group (enumerated as Chis150) after 6, 24 and 48 h of fermentation in pH-controlled batch culture systems. Increases/decreases refer to the inoculum of the negative control. Samples: A, negative control healthy donors; B, healthy donors + CB; C, healthy donors + GFB; D, negative control coeliac donors; E, coeliac donors + CB; F, coeliac donors + GFB. CB, control bread; GFB, gluten-friendly bread.

	FISH (log cells mL^-1^)	Homogeneous group		
6 h		I	II	III
Sample				
B	-0.27			
A	-0.18			
C	-0.10			
D	0.15			
F	0.17			
E	0.32			
**24 h**				
C	-0.16			
F	-0.12			
A	-0.08			
B	-0.03			
E	0.07			
D	0.10			
**48 h**				
A	-0.31			
C	-0.23			
B	0.06			
D	0.17			
F	0.19			
E	0.22			

The lactic acid bacteria exhibited a characteristic trend over time, as reported in Table 5. The 6 h fermentation caused a decrease in the numbers of *Lactobacillus/Enterococcus* spp. in samples E and F (0.57–0.64 log CFU mL^-1^). After 24 h, the decrease was again observed in sample E but not in sample F, which exhibited a trend similar to the samples from healthy donors (no decrease in the cell count). After 48 h, the samples were distributed in a continuous way, and sample F was included in the group of healthy donors, as well as in the same group as sample E, suggesting that the shift observed after 24 h of fermentation was reversible. The statistical outputs for *E*. *rectale* pinpoint a constant distribution, without significant differences amongst the different samples (S4 Table).

**Table 5:** One-way ANOVA (P<0.05) and homogeneous groups based on FISH data for *Lactobacillus/Enterococcus* (enumerated with Lab158) after 6, 24 and 48 h of fermentation in pH-controlled batch culture systems. Increases/decreases refer to the inoculum of the negative control. Samples: A, negative control healthy donors; B, healthy donors + CB; C, healthy donors + GFB; D, negative control coeliac donors; E, coeliac donors + CB; F, coeliac donors + GFB. CB, control bread; GFB, gluten-friendly bread.

	FISH (log cells mL^-1^)	Homogeneous group		
6 h		I	II	III
Sample				
F	-0.64			
E	-0.57			
D	-0.33			
C	-0.12			
A	0.00			
B	0.04			
**24 h**				
E	-0.59			
F	0.01			
D	0.02			
A	0.17			
C	0.27			
B	0.29			
**48 h**				
E	-0.53			
D	-0.29			
F	-0.02			
A	0.14			
B	0.19			
C	0.30			

The same approach was used to analyse the SCFA profiles (Tables 6, 7 and 8). The amount of SCFAs generally had a discrete distribution with well-defined statistical groups and significant differences. Acetate increased by 174 mM in sample B after 24 h, whereas the increase was 43–62 mM in the other samples. A similar trend was observed at the end of the assay, although a further increase was found (Table 6). The increase in propionic acid was slight in the samples from healthy donors (after 24 and 48 h), whereas the content of this acid increased by 23–37 mM in the samples from coeliac donors (Table 7). After 24 h, butyric acid increased by 17 mM in the negative control D, which experienced the highest increase, followed by the other two samples from the coeliac donors (E, 7.6 mM; F, 4.1 mM). The results after 48 h revealed an interesting output; sample E experienced a further increase in butyric acid of 3 mM, and an increase was found in samples A (2 mM), B (4 mM) and C (6.58 mM); sample F showed a profile similar to the samples from healthy donors, with a net increase in butyric acid of 4.28 mM (Table 8).

**Table 6:** One-way ANOVA (P<0.05) and homogeneous groups based on acetate after 24 and 48 h of fermentation in pH-controlled batch culture systems. Increases/decreases refer to the inoculum of the negative control. Samples: A, negative control healthy donors; B, healthy donors + CB; C, healthy donors + GFB; D, negative control coeliac donors; E, coeliac donors + CB; F, coeliac donors + GFB. CB, control bread; GFB, gluten-friendly bread.

	Acetate (mM)	Homogeneous groups					
24 h		I	II	III	IV	V	VI
Sample							
C	43.37						
A	45.39						
D	52.36						
F	52.38						
E	62.30						
B	174.10						
**48 h**							
A	43.86						
F	52.96						
E	57.66						
D	61.84						
C	62.46						
B	258.47						

**Table 7:** One-way ANOVA (P<0.05) and homogeneous groups based on propionate after 24 and 48 h of fermentation in pH-controlled batch culture systems. Increases/decreases refer to the inoculum of the negative control. Samples: A, negative control healthy donors; B, healthy donors + CB; C, healthy donors + GFB; D, negative control coeliac donors; E, coeliac donors + CB; F, coeliac donors + GFB. CB, control bread; GFB, gluten-friendly bread.

	Propionate (mM)	Homogeneous groups					
24 h		I	II	III	IV	V	VI
Sample							
B	-4.31						
A	-0.76						
C	0.97						
D	23.16						
E	31.19						
F	32.71						
48 h							
A	0.39						
B	1.72						
C	4.69						
D	22.59						
F	37.14						
E	37.46						

**Table 8:** One-way ANOVA (P<0.05) and homogeneous groups based on butyrate after 24 and 48 h of fermentation in pH-controlled batch culture systems. Increases/decreases refer to the inoculum of the negative control. Samples: A, negative control healthy donors; B, healthy donors + CB; C, healthy donors + GFB; D, negative control coeliac donors; E, coeliac donors + CB; F, coeliac donors + GFB. CB, control bread; GFB, gluten-friendly bread.

	Butyrate (mM)	Homogeneous group					
24 h		I	II	III	IV	V	VI
Sample							
**C**	-3.63						
**B**	-0.39						
**A**	2.43						
**F**	4.07						
**E**	7.59						
**D**	17.42						
**48 h**							
**A**	2.25						
**B**	4.02						
**F**	4.28						
**C**	6.59						
**E**	10.67						
**D**	15.04						

### Changes in the bacterial populations and metabolic profile with *in vitro* batch culture fermentation: a multivariate approach

**Fig 4:**
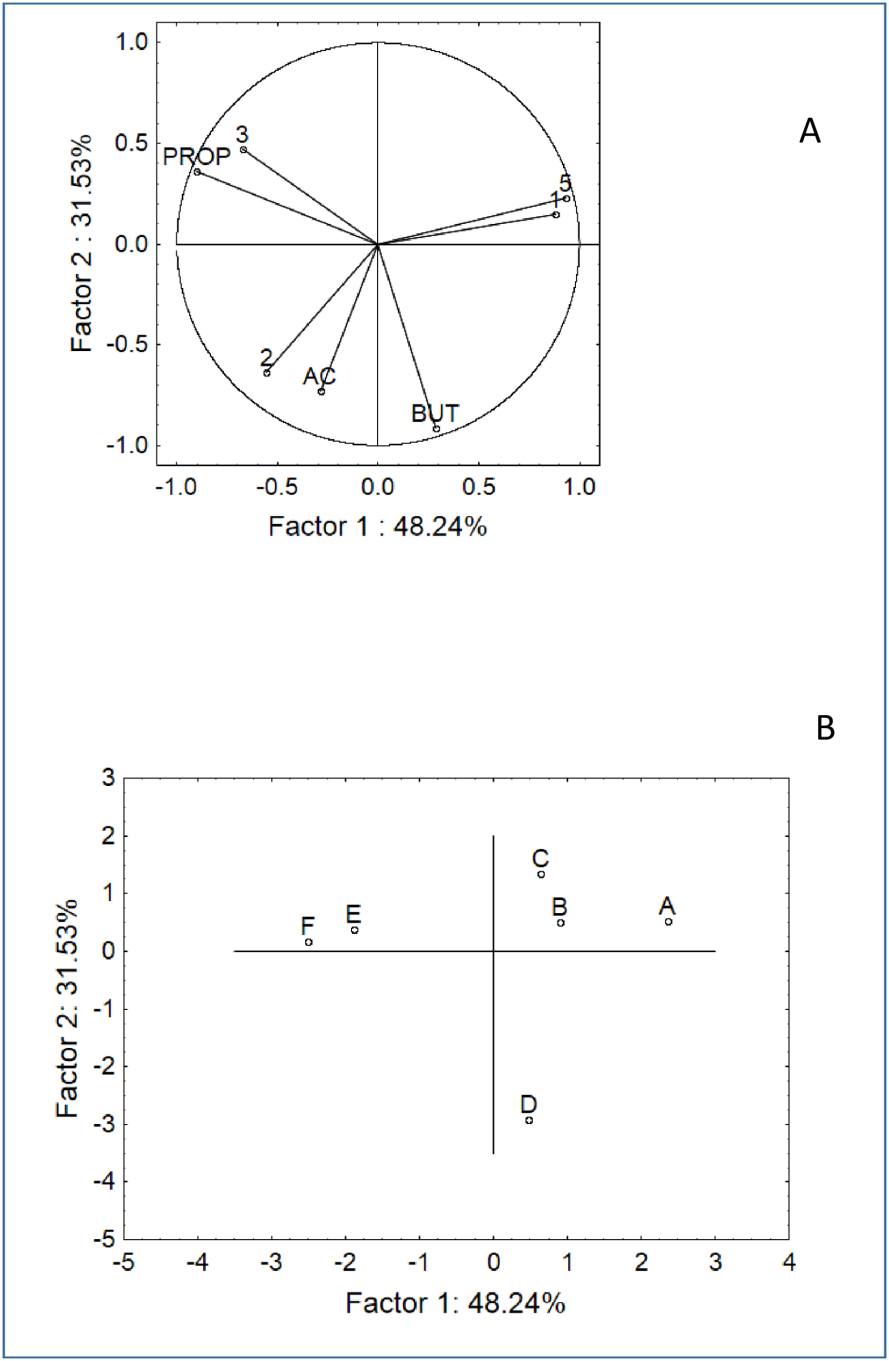
PCA based on the SCFA and FISH data after 6 h of fermentation. A) Variable projection; B) Case projection. Samples: A, negative control healthy donors; B, healthy donors + CB; C, healthy donors + GFB; D, negative control coeliac donors; E, coeliac donors + CB; F, coeliac donors + GFB. CB, control bread; GFB, gluten-friendly bread. Variables: 1, bifidobacteria; 2, *E*. *rectale-C*. *coccoides*; 3, *Bacteroides*/*Prevotella*; 5, *Lactobacillus/Enterococcus*; AC, acetate; BUT, butyrate; PROP, propionate.

**Fig 5:**
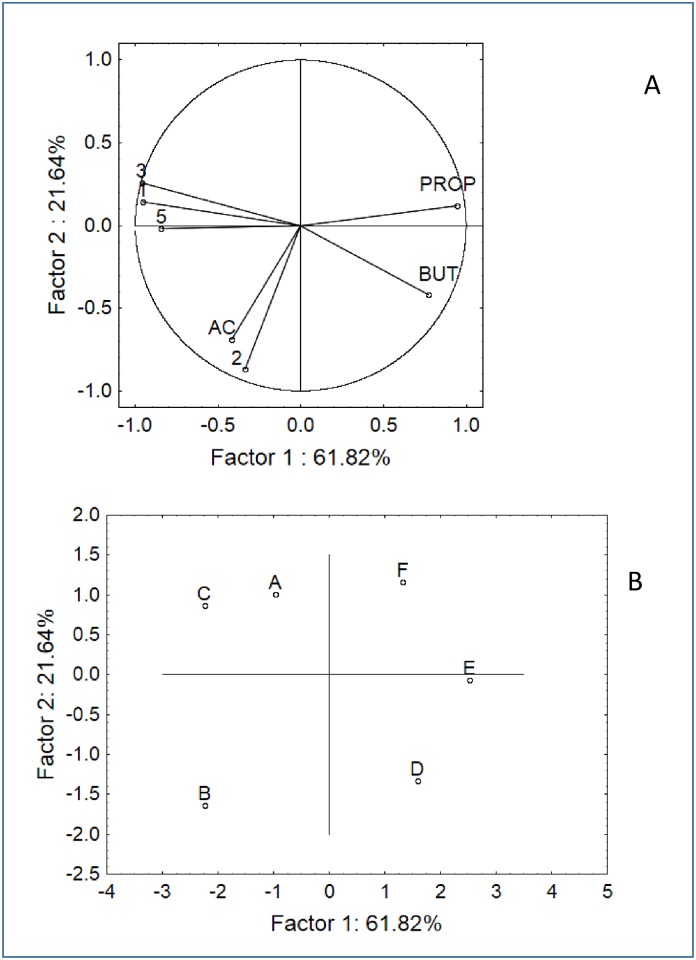
PCA based on the SCFA and FISH data after 24 h of fermentation in pH-controlled batch cultures. A) Variable projection; B) Case projection.

**Fig 6:**
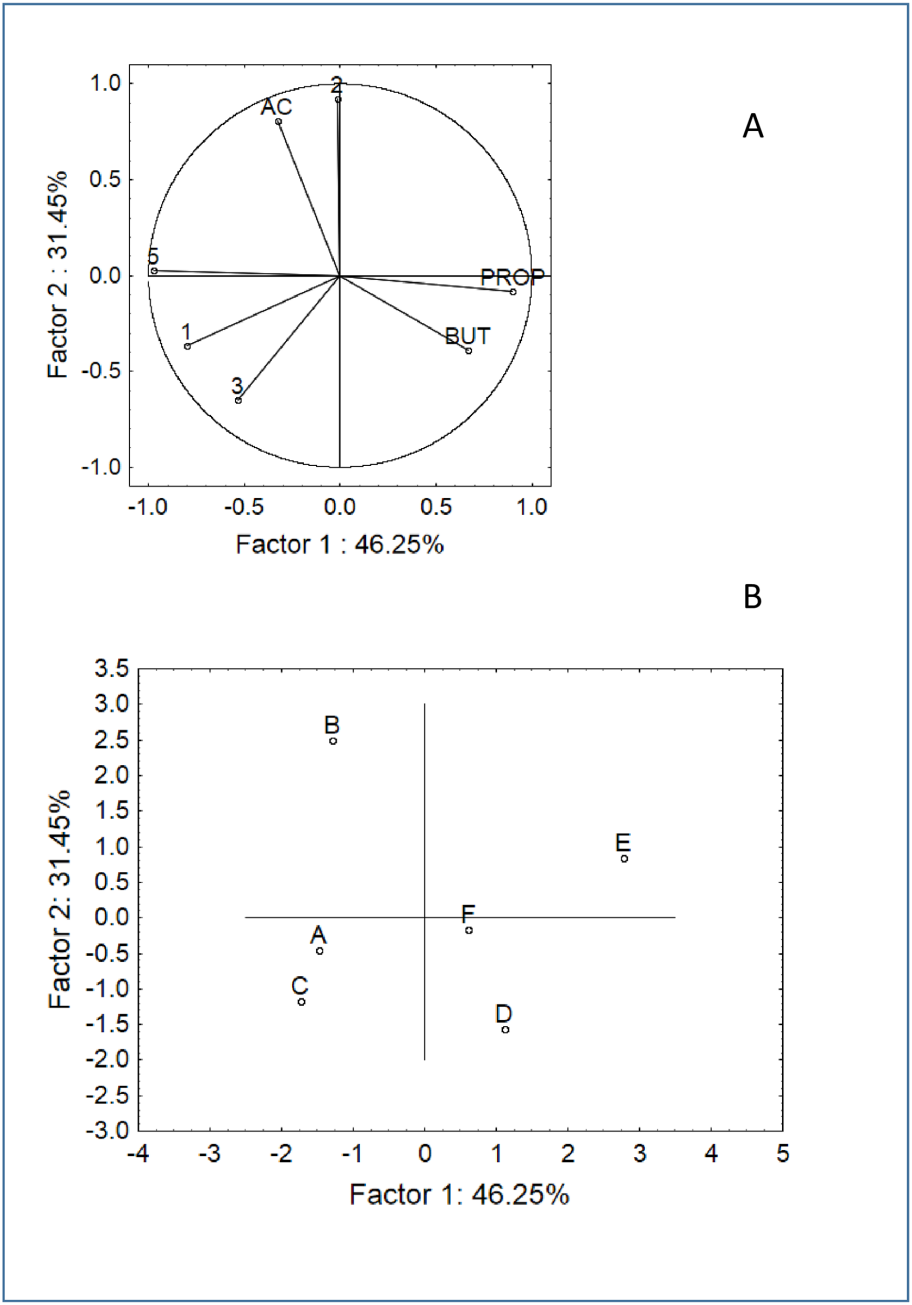
PCA based on the SCFA and FISH data after 48 h of fermentation in pH-controlled batch cultures. A) Variable projection; B) Case projection.

The results from the batch cultures were analysed through PCA. SCFA and FISH data were all used as input, except for the data on the *C*. *hystolyticum* and *E*. *rectale–C*. *coccoides* groups due to the results of one-way ANOVA (no significant differences). Three analyses were run in order to assess the factorial distribution of the samples as a function of the origin (coeliac or healthy donors), supplementation (CB or GFB) and time (6, 24, and 48 h), as shown in Figs 4–6. For each analysis, we reported both the variable (Figs 4A, 5A and 6A) and case distribution (Figs 4B, 5B and 6B); however, we did not use PCA to discuss why and for which type of variable the samples were different (this topic was addressed in the previous section). We used PCA to graphically estimate the similarity or dissimilarity amongst the samples. After 6 h, 2 statistical groups could be determined in the multi-factorial space, i.e., the first group including the samples from healthy donors (A, B, C) and the second group including the samples from coeliac donors supplemented with CB or GFB (E and F). The negative control of this latter group was an outsider and placed in a different region of the factorial space (Fig 4).

After 24 h, the space distribution drastically changed, and many samples experienced a shift in the plot (Fig 5); the group composed of healthy donors was divided in two sub-groups because sample B had moved from a different region of the space. A split was also found for the samples from coeliac donors: both E and F moved away from the negative control, but F moved in the upper region of the space towards samples A and C. A similar distribution was found after 48 h, except that samples B and E were in the positive quadrants of factor 2 (upper region) and the other samples were in the negative region (Fig 6).

## Discussion

The role of gut microbiota in health and well-being has been extensively reviewed [30]. Lifestyle, diet, life stage and some pathologies can strongly affect the qualitative and quantitative composition of gut microbiota [31,32]. Coeliac people possess altered gut microbiota; in addition, many researchers pinpointed that dysbiosis dramatically impacts the host physiology [33].

The background of this research was a patent [14,15] focused on the use of microwave for the detoxification of gluten. The safety of this technology was preliminary assessed on peripheral blood mononuclear cells (PBMC) stimulated with phytohaemagglutinin (PHA). The patent was further improved in terms of combination of microwave power, treatment time and rest time [18]; however, the effect of this approach on the microbiota of the gut has been never addressed and this topic has been investigated in the present paper.

Firstly, we aimed to determine whether GFB modifies the qualitative-quantitative composition of gut microbiota via a two-step plan. The first phase relied on the evaluation of the death decay of selected strains and focused on the eventual change of shape or kinetics of the characteristic curve of each microorganism. For this step, the Weibull function pinpointed some interesting results. Namely, the Weibull equation possesses two fitting parameters (δ and p for the first reduction time and the shape parameter, respectively), which take into account two different phenomena of death kinetics, i.e., the prolongation of the shoulder phase (a benefit in the first step of the kinetics) and tailing (i.e., prolonged survival). As an additional tool, we used a slightly modified Weibull equation to evaluate death (or survival time). This last parameter is generally affected by the shoulder and tail, as well as the death rate. Supplementation with GFB affected neither the first reduction time nor the shape parameter of *L*. *acidophilus* but exerted a significant effect on the death time. GFB probably did not induce resistance in cells but lowered the death rate by exerting a protective effect. However, this effect was quite different from the protection exerted by some prebiotics. For example, fructooligosaccharides and inulin induced a prolonged tail as a result of a general stress response mechanism due to starvation [34,35], whereas GFB resulted in a reduction of the death kinetics but not a tail effect.

The lowering of death rate in the bacterial curve, as well as growth enhancement, is generally the result of a protective compound and has observed previously in some cell-free filtrates or bifidogenic factors. The effects of these factors were variable and described as growth enhancement due to membrane permeability, combating cell aging, etc. [36,37].

The high temperature generated by the microwave treatment and applied to the hydrated wheat caryopses for a short period of time to detoxify gluten may break the hydrogen bonds between the glutamine residues in proteins when in their native form in protein bodies, inducing a rearrangement of the secondary and tertiary structure of the gluten protein, with a different spatial conformation of the toxic sequences [16]. We also postulated that the rearrangement of some of the gluten protein structure involves the exposure of positive charges [18]. Positive molecules, namely cationic peptides, can exert a strong antibacterial effect because they supposedly act at the cytoplasmic membrane, leading to permeabilisation and eventually membrane disruption. Arginine and lysine residues play a major role in this process [38]. Moreover, the interaction of antimicrobial peptides with anionic membrane phospholipids is a key factor in killing bacteria [38]. To date, it is not clear whether the outer membrane of Gram-negative bacteria can exert a positive or negative effect. Our results suggest that the outer membrane did not play a role due to the significant antibacterial effect on both *Salmonella* Typhimurium and *S*. *aureus*.

Teichoic acid can act negatively on cationic peptides because they have a negative charge, catch positive molecules and decrease their potential towards cells [38]. This idea could partially explain how GFB did not exert a negative effect on lactobacilli and bifidobacteria but did for *S*. *aureus*, and this difference might be the result of the unique structure of the cell wall and teichoic acids in lactobacilli, as described by Chapot-Chartier and Kulakauskas [39].

After this preliminary evaluation in strictly controlled conditions, we moved to a complex ecosystem to assess whether GFB can affect the evolution of heterogeneous microbiota. Two variables were assessed: the type of bread (CB and GFB) and the subject (coeliac or healthy people). The experiments were performed using 6 different subjects, and a negative control (batch culture inoculated with faecal microbiota but not supplemented with any type of bread) was also added for each subject. In addition, we also assumed that the faecal microbiota could experience a qualitative-quantitative change *per se* (decrease or increase without supplementation due to a “donor effect”) or after bread supplementation (change due a “bread effect”). Thus, we used a static approach to standardize the data to the negative control at each sampling time. For each group (healthy subjects, A-C; coeliac subjects, D-F), the standardized values of the negative controls (A and D) showed the donor effect not related to bread supplementation. Therefore, a negative value pinpointed that bread supplementation caused a decrease in the viable count, whereas a positive value highlighted an increase.

Some groups were chosen as tests to assess the effect of CB and GFB, such as lactobacilli, bifidobacteria, *Bacteroidetes*, eubacteria and clostridia. Bifidobacteria can produce vitamins (e.g., K, B_12_, biotin, folate, thiamine) [31]. The synthesis of secondary bile acids is mediated *via Lactobacillus* spp. and *Bifidobacterium* spp. [31]. Moreover, *Bifidobacterium* spp. can also help prevent pathogenic infection through the production of acetate [40]. To date, the role of *Bacteroides* is controversial: some authors have postulated a positive impact, whereas other researchers have found a strong correlation of these microorganisms with CD [41,42] and a possible role in the pro-inflammatory response [42], mucin degradation and increased permeability of the small intestine [43,44]. *E*. *rectale* (now reclassified as *Agathobacter rectalis*) [45] is generally related to bifidobacteria, as it produces butyrate from acetate [46], with a beneficial effect on the host.

The most valuable results were found for lactobacilli and bifidobacteria. In fact, the standardised values of lactobacilli from coeliac donors after 6 h were negative, suggesting that the lactobacilli population suffered a type of stress that was enhanced by bread supplementation. The shift from negative to positive values in coeliac subjects in the presence of GFB (sample F) suggests that the supplementation suddenly interrupted this stress and beneficially modulated the microbiota composition. However, this effect was reversible because the samples experienced a partial shift after 48 h, suggesting that a prolonged and a beneficial effect could also be the result of prolonged supplementation. The experimental data from the batch cultures also confirmed the ability of GFB to promote the growth of *Lactobacillus* spp. The shift was much stronger in coeliac donors probably due the unbalanced microbiota composition compared to healthy subjects.

Differently from the screening, GFB exerted a positive effect on the bifidobacteria numbers of coeliac donors, although the effect was found after only 48 h. This result, along with the death kinetics data in saline solution, suggests that bifidobacteria probably require prolonged supplementation.

The same approach was used to model and analyse SCFA profiles. There is growing recognition of the role of SCFAs in immune function and inflammation in tissues [47]. Moreover, SCFAs can act as key sources of energy for colorectal tissues and bacteria and promote cellular mechanisms that maintain tissue integrity [48–50]. The data were quite variable, and bread supplementation did not exert a clear effect. To better understand this scenario, we decided to combine the SCFA data with the viable counts and pinpoint the changes at a global level.

Thus, the last statistical analysis (PCA) pinpointed a change in the ecosystem, and this effect was clearly distinguishable after 24 hours for sample F (coeliac donor+GFB), which moved from the region of coeliac people to healthy subjects. Thus suggests that GFB can cause a change in the whole ecosystem and exert a key role in the fight against the dysbiosis in coeliac people.

This study provides new insights into the role of GFB on the qualitative-quantitative modulation of microbiota in simple or complex systems. The first step pinpointed that GFB has an important role in the prolongation of the survival of *L*. *acidophilus* and the antibacterial effect towards *S*. *aureus* and *Salmonell*a Typhimurium. In a complex ecosystem, such as gut microbiota, GFB induced a beneficial modulation in terms of bifidogenic effects and on the growth of lactobacilli. Moreover, a final multivariate approach combining both the viable count and SCFA profile suggested that GFB causes a shift in the whole ecosystem. Therefore, this paper provides findings supporting the utilization of GFB to modulate the composition and metabolic profile of the intestinal microbiota in coeliac individuals. The applicability of such changes remains to be shown in a 3-stage continuous *in vitro* colonic model and an *in vivo* trial.

## Supporting Information

S1 Fig Cell counts (mean±SD) of *Bifidobacterium* spp. (Bif164), *Clostridium hystoliticum*/*perfringens* group (Chis150) and lactobacilli/enterococci (Lab158) in pH-controlled batch systems at the beginning and after 6, 24 and 48 h of fermentation. A, negative control healthy donors; B, healthy donors + control bread; C, healthy donors + gluten friendly bread; D, negative control coeliac donors; E, coeliac donors + control bread; F, coeliac donors + gluten friendly bread. The data were preliminary analyzed to exclude the outliers.
(DOCX)

S2 Fig Concentration (mM) of acetate, propionate and butyrate in pH-controlled batch systems at the beginning and after 6, 24 and 48 h of fermentation. A, negative control healthy donors; B, healthy donors + control bread; C, healthy donors + gluten friendly bread; D, negative control coeliac donors; E, coeliac donors + control bread; F, coeliac donors + gluten friendly bread. The data were preliminary analyzed to exclude the outliers.
(DOCX)

S1 Table Fitting parameters of the Weibull equation for the death kinetics of *B*. *animalis* subsp. *lactis* (mean values ± SE). CB, control bread; GFB, gluten-friendly bread.
(DOCX)

S2 Table Cell counts of *L*. *acidophilus* and *B*. *animalis* subsp. *lacti*s (mean±SD) (log CFU mL^-1^) in a saline solution supplemented with either control (CB) or gluten-friendly bread (GFB) (0.8 and 5.0 g L^-1^) (incubation at 37°C for 24 h). The letters indicate the significant differences within each column (one-way ANOVA and Tukey’s test, P<0.05).
(DOCX)

S3 Table One-way ANOVA (P<0.05) and homogeneous groups based on FISH data for *Bacteroides/Prevotella* group (enumerated as Bac303) after 6, 24 and 48 h of fermentation in pH-controlled batch culture systems. Increases/decreases refer to the inoculum of the negative control. Samples: A, negative control healthy donors; B, healthy donors + CB; C, healthy donors + GFB; D, negative control coeliac donors; E, coeliac donors + CB; F, coeliac donors + GFB. CB, control bread; GFB, gluten-friendly bread.
(DOCX)

S4 Table One-way ANOVA (P<0.05) and homogeneous groups based on FISH data for *E*. *rectale/C*. *coccoides* group (enumerated as Erec482) after 6, 24 and 48 h of fermentation in pH-controlled batch culture systems. Increases/decreases refer to the inoculum of the negative control. Samples: A, negative control healthy donors; B, healthy donors + CB; C, healthy donors + GFB; D, negative control coeliac donors; E, coeliac donors + CB; F, coeliac donors + GFB. CB, control bread; GFB, gluten-friendly bread.
(DOCX)
