# Supplementary material for: Impact of Gluten-Friendly Bread on the Metabolism and Function of In Vitro Gut Microbiota in Healthy Human and Coeliac Subjects
Source: PLoS One. 2016 Sep 15;11(9):e0162770. doi: 10.1371/journal.pone.0162770 (PMC5025162; doi:10.1371/journal.pone.0162770)
Supplement: S1 Fig — A, negative control healthy donors; B, healthy donors + control bread; C, healthy donors + gluten friendly bread; D, negative control coeliac donors; E, coeliac donors + control bread; F, coeliac donors + gluten friendly bread. The data were preliminary analyzed to exclude the outliers. (DOCX) [file pone.0162770.s001.docx]

**A**

B

C
