# Supplementary material for: Impact of Gluten-Friendly Bread on the Metabolism and Function of In Vitro Gut Microbiota in Healthy Human and Coeliac Subjects
Source: PLoS One. 2016 Sep 15;11(9):e0162770. doi: 10.1371/journal.pone.0162770 (PMC5025162; doi:10.1371/journal.pone.0162770)
Supplement: S1 Table — CB, control bread; GFB, gluten-friendly bread. (DOCX) [file pone.0162770.s003.docx]

| **Sample** | **log N_0_*** | **δ** | **p** | **R** |
| --- | --- | --- | --- | --- |
| CB 0.4 g L^-1^ | 8.09±0.14 | 16.98±0.90 | 1.42±0.11 | 0.991 |
| GFB 0.4 g L^-1^ | 8.18±0.13 | 17.03±2.06 | 1.44±0.14 | 0.999 |
| CB 0.8 g L^-1^ | 8.29±0.12 | 17.23±2.00 | 1.34±0.08 | 0.992 |
| GFB 0.8 g L^-1^ | 8.06±0.14 | 17.27±0.35 | 1.25±0.17 | 0.995 |

*log N_0_, initial cell count (log CFU mL^-1^); δ, first reduction time (h); p, shape parameter.
