# Supplementary material for: Impact of Gluten-Friendly Bread on the Metabolism and Function of In Vitro Gut Microbiota in Healthy Human and Coeliac Subjects
Source: PLoS One. 2016 Sep 15;11(9):e0162770. doi: 10.1371/journal.pone.0162770 (PMC5025162; doi:10.1371/journal.pone.0162770)
Supplement: S2 Table — The letters indicate the significant differences within each column (one-way ANOVA and Tukey’s test, P<0.05). (DOCX) [file pone.0162770.s004.docx]

|  | *L. acidophilus* | *B. animalis* subsp. *lactis* |
| --- | --- | --- |
| Inoculum | 8.07±0.02A | 8.70±0.07A |
| 0.8 g L^-1^ |  |  |
| CB | 7.14±0.17B | 8.54±0.05A |
| GFB | 7.40±0.11B | 8.51±0.07A |
| 5.0 g L^-1^ |  |  |
| CB | 7.57±0.14A,B | 9.30±0.04B |
| GFB | 7.73±0.07 A,B | 9.25±0.03B |
