# Supplementary material for: Impact of Gluten-Friendly Bread on the Metabolism and Function of In Vitro Gut Microbiota in Healthy Human and Coeliac Subjects
Source: PLoS One. 2016 Sep 15;11(9):e0162770. doi: 10.1371/journal.pone.0162770 (PMC5025162; doi:10.1371/journal.pone.0162770)
Supplement: S3 Table — Increases/decreases refer to the inoculum of the negative control. Samples: A, negative control healthy donors; B, healthy donors + CB; C, healthy donors + GFB; D, negative control coeliac donors; E, coeliac donors + CB; F, coeliac donors + GFB. CB, control bread; GFB, gluten-friendly bread. (DOCX) [file pone.0162770.s005.docx]

|  |  | **Homogeneous groups** | | | |
| --- | --- | --- | --- | --- | --- |
| **Sample** | **FISH (log cells mL^-1^)** | **I** | **II** | **III** | **IV** |
| **6 h** |  |  |  |  |  |
| **A** | -0.09 |  |  |  |  |
| **D** | -0.04 |  |  |  |  |
| **E** | 0.08 |  |  |  |  |
| **B** | 0.11 |  |  |  |  |
| **C** | 0.13 |  |  |  |  |
| **F** | 0.18 |  |  |  |  |
| **24 h** |  |  |  |  |  |
| **D** | -0.19 |  |  |  |  |
| **E** | -0.17 |  |  |  |  |
| **F** | 0.03 |  |  |  |  |
| **A** | 0.41 |  |  |  |  |
| **B** | 0.43 |  |  |  |  |
| **C** | 0.64 |  |  |  |  |
| **48 h** |  |  |  |  |  |
| **B** | -0.33 |  |  |  |  |
| **F** | -0.31 |  |  |  |  |
| **E** | -0.31 |  |  |  |  |
| **D** | -0.19 |  |  |  |  |
| **C** | -0.11 |  |  |  |  |
| **A** | -0.03 |  |  |  |  |
